# Classical-Equivalent Bayesian Portfolio Optimization for Electricity Generation Planning †

**DOI:** 10.3390/e20010042

**Published:** 2018-01-10

**Authors:** Hellinton H. Takada, Julio M. Stern, Oswaldo L. V. Costa, Celma O. Ribeiro

**Affiliations:** 1 Quantitative Research, Itaú Asset Management, São Paulo 04538-132, Brazil; 2 Institute of Mathematics and Statistics, University of São Paulo, São Paulo 05508-090, Brazil; 3 Polytechnic School, University of São Paulo, São Paulo 05508-010, Brazil

**Keywords:** statistics, inference methods, energy analysis, policy issues, 02.50.-r, 02.50.Tt, 88.05.-b, 88.05.Jk

## Abstract

There are several electricity generation technologies based on different sources such as wind, biomass, gas, coal, and so on. The consideration of the uncertainties associated with the future costs of such technologies is crucial for planning purposes. In the literature, the allocation of resources in the available technologies has been solved as a mean-variance optimization problem assuming knowledge of the expected values and the covariance matrix of the costs. However, in practice, they are not exactly known parameters. Consequently, the obtained optimal allocations from the mean-variance optimization are not robust to possible estimation errors of such parameters. Additionally, it is usual to have electricity generation technology specialists participating in the planning processes and, obviously, the consideration of useful prior information based on their previous experience is of utmost importance. The Bayesian models consider not only the uncertainty in the parameters, but also the prior information from the specialists. In this paper, we introduce the classical-equivalent Bayesian mean-variance optimization to solve the electricity generation planning problem using both improper and proper prior distributions for the parameters. In order to illustrate our approach, we present an application comparing the classical-equivalent Bayesian with the naive mean-variance optimal portfolios.

## 1. Introduction

During the time, the mankind has developed several electricity generation technologies based on different primary sources such as wind, biomass, gas, coal, nuclear, and so on. Evidently, each technology has associated costs, sustainability and security of supply characteristics, efficiency and environmental concerns. According to the United States Environmental Protection Agency, the different primary energy sources are organized by conventional power, such as oil, natural gas, coal and nuclear; renewable energy, such as large hydropower and municipal solid waste; and green power, such as wind, solar, biomass, geothermal, biogas and low-impact hydropower. In particular, the low-impact hydropower is the use of hydroelectric power on a scale suitable for local community and industry, or to contribute to distributed generation in a regional electricity grid, with a lower negative environmental impact compared to the large hydropower. In terms of less environmental impacts, the conventional power sources are the least beneficial and the green power sources are the most beneficial.

The worldwide demand for energy has been increasing over the last decades and it will continue to grow [1]. Consequently, for both countries and companies, the long-term planning of the electricity generation infrastructure is of utmost importance. Actually, it should be part of the central objectives of any energy policy. The achievement of an optimal designed electricity generation infrastructure bends towards a more balanced portfolio allocation among the different available technologies. In addition, it is also important to distinguish in the planning process the already existing electricity producing plants with maintenance costs from the ones desired to be built. Economically, drastic changes of the current electricity investment allocations are not feasible. In this paper, our model distinguishes the costs of the already existing from the costs of the prospective or desired to be built plants.

The United States Energy Information Administration not only has the history of the average annual maintenance, operational, and fuel costs for existing power plants by energy source or major fuel types, but also projections for electricity generation costs [2]. However, even so, the costs have a significant uncertainty. For instance, future control on CO_2_ emission and the corresponding mechanisms will surely impact the electricity generation costs. Precisely, the future price of an emitted ton of CO_2_ is uncertain and this uncertainty should be considered in the planning process. Consequently, electricity generation policies solely relying on the evolution of historical average costs of electricity generation technologies are unsatisfactory. The careful consideration of the uncertainties associated with the current and the prospective costs of such technologies is fundamental for planning purposes.

Considering the costs as random variables, in the literature, the allocation of resources in the available electricity generation technologies has been solved as a mean-variance optimization problem using the expected values and covariance matrix of the technology costs in megawatt hours (see, for instance, [3,4,5,6]). The mean-variance optimization, introduced by Markowitz [7], was the first mathematical formalization of investment diversification and it is part of the modern portfolio theory (MPT). The mean-variance optimized portfolios compose the called efficient frontier, a set of portfolios that dominate all other feasible portfolios in terms of their mean and variance tradeoff. Clearly, in the MPT, the random variables of interest are the returns of the risky assets instead of the costs of the technologies.

In practice, the expected values and the covariance matrix of the electricity generation technology costs for a future time horizon are not exactly known. The use of only historical data to estimate the expected values and covariance matrix is a naive approach because the past will not necessarily repeat in the future. Noticeably, the usefulness of the allocations obtained from the mean-variance optimization depends on the preciseness of such parameters. For instance, in the MPT context, it was shown in [8] that minor changes in the expected values of returns can produce major changes in asset allocation decisions. Consequently, several robust versions of the mean-variance optimization were proposed in the MPT literature to consider uncertainties on the expected returns and covariance matrix (see, for instance, [9,10,11]).

There are many published research on uncertainty analysis using Bayesian methods for the energy industry. For instance, the application of Bayesian networks in the renewable energy area to deal with storage, smart grids and assessment are ample (for a complete survey, see [12]). Bayesian network is a technique used to deal with problems with uncertainty [13,14]. The related literature is diverse including a building occupants representation model for energy efficiency using Bayesian networks [15] to a Bayesian framework for power network planning using statistical emulators [16]. A model and the computer program used to implement it are referred to as a simulator and an emulator is a statistical approximation of a simulator [17,18]. Basically, the uncertainty in the inputs of the models is represented as a probability distribution in a Bayesian framework.

Particularly in [19], in the electricity planning context using MPT, it was presented a robust portfolio optimization approach to deal with uncertainties in the input parameters. The uncertainty in the robust portfolio optimization approach is represented by an uncertainty set for the input parameters. In [19], the uncertainty sets considered were the box, the ellipsoidal, the lower and the upper bounds, and the convex polytopic. However, the energy planning process is very complex and involves other concerns such as sustainability, resiliency, availability, reliability, efficiency, safety and security of the generation technologies. Such concerns add not only additional uncertainty in the costs of such technologies but also beliefs that come from technology specialists. Actually, it is usual to have the participation of specialists in the electricity generation technologies of interest in the electricity planning processes.

Undoubtedly, a natural way of conducting a comprehensive planning process is to take into account the available data together with the prior experience of the participant specialists. Bayesian approaches treat the probability distributions themselves as uncertain and subject to updates as new information arrives. Consequently, the Bayesian approach has been successfully applied in the MPT context to take into account not only the beliefs of the investors but also the uncertainties in the expected returns and the correspondent covariance matrix (see, for instance, [8,20,21]). The Bayesian mean-variance portfolio optimizations consider both the estimation uncertainty and the specialist prior information. In a few words, the prior probability represents the beliefs of the investment specialists, the probability update represents the incorporation of the available data in the model and the predictive probability represents the updated beliefs of the specialists using the available data.

In the literature, there are different existent Bayesian approaches to deal with the parameter uncertainty in the context of MPT (for instance, see [22,23,24,25,26,27,28,29]). Historically, the initial applications of Bayesian approaches in 1970s were based on improper or data-based priors [30]. The Bayesian approaches based on improper priors usually give comparable results to the classical methods and the difference arises when some risky assets have longer historical data than others [31]. Then, trying to incorporate prior information into the asset allocation model, the Black–Litterman model was introduced using a Bayesian approach to include investors views and equilibrium relations in the portfolio allocation [8]. The main difficulty to apply Black-Litterman model in practice is that it requires the investors views as inputs and, usually, they are not publicly available. Other studies are centering prior beliefs around values implied by asset pricing theories [32,33] or using investment objectives to obtain useful priors [34].

In this paper, our objective is the introduction of the classical-equivalent Bayesian portfolio optimization to electricity generation planning. The main contribution of our Bayesian approach is the possibility to take into account both the estimation uncertainty and the specialists’ information at the same time in the energy planning process. In the next section, we give a brief review of the classical mean-variance optimization with the basic notation and fundamental concepts. Then, we present the classical-equivalent Bayesian approach using both improper and proper priors. In addition, for illustration purposes, we compare the classical-equivalent Bayesian optimal portfolios with the classical mean-variance optimal portfolios using the same data from [19,35]. Finally, we present some final comments about our proposed approach and suggestions for future research at the end of the paper.

## 2. Classical or Naive Mean-Variance Approach

Traditionally, the classical or naive mean-variance optimization assumes that cost and risk, the last one measured as the portfolio volatility, are known when making portfolio allocation decisions. For that reason, a rational planner would prefer a portfolio with a lower expected cost for a given level of risk. Alternatively, a preferred portfolio is the one that minimizes risk for a given expected cost level. The set of portfolios that are optimal is called the efficient frontier. No rational planner would select a portfolio lying above the efficient frontier, since that would mean accepting a higher cost for the same amount of risk as an efficient portfolio. Similarly, it would mean accepting greater risk for the same expected cost as an efficient portfolio.

Following [19,35], it is important to distinguish in the planning process an already existing electricity producing plant using technology *i*, with random cost $C_{i}^{e}$ in USD/MWh, from a prospective idea of building a new plant using *i*, with random cost $C_{i}^{p}$ in USD/MWh. In practice, substantial changes of the current electricity investment allocations are not feasible and the maintenance costs of existing plants are different from the implementation costs of new plants. The random vectors of costs for existing plants and prospective ideas of building new plants when there are *N* different technologies are given by

(1) $$C^{e}\equiv {\left(C_{1}^{e}\;C_{2}^{e}\;\ldots \;C_{N}^{e}\right)}^{\prime }\;\mathrm{and}\;C^{p}\equiv {\left(C_{1}^{p}\;C_{2}^{p}\;\ldots \;C_{N}^{p}\right)}^{\prime },$$

respectively. It is also usual to assume that the random costs are multivariate normal

(2) $$C^{e}\left|\mu^{e},\Sigma^{e}∼N\left(\mu^{e},\Sigma^{e}\right)\;\mathrm{and}\;C^{p}\right|\mu^{p},\Sigma^{p}∼N\left(\mu^{p},\Sigma^{p}\right),$$

where $\mu^{e}={\left(\mu_{i}^{e}\right)}_{N\times 1}$ and $\mu^{p}={\left(\mu_{i}^{p}\right)}_{N\times 1}$ are mean vectors and $\Sigma^{e}$ and $\Sigma^{p}$ are N×N covariance matrices. The means $\mu_{i}^{e}$ and $\mu_{i}^{p}$ are different because maintenance costs are different from the costs of building a new plant. Additionally, the risk or standard deviation of maintenance $\sigma_{i}^{e}$ is also different from the risk or standard deviation of building a new plant $\sigma_{i}^{p}$. However, since the technology is the same, the correlation between $C_{i}^{e}$ and $C_{i}^{p}$ is equal to $\rho_{C_{i}^{e},C_{i}^{p}}=1$. Thus, we can write almost surely (with probability 1) that (see Proposition 1.1.2 from [36])

(3) $$C_{i}^{e}=\frac{\sigma_{i}^{e}}{\sigma_{i}^{p}}\left(C_{i}^{p}-\mu_{i}^{p}\right)+\mu_{i}^{e}.$$

Essentially, Equation (3) says that the source of uncertainty for both $C_{i}^{e}$ and $C_{i}^{p}$ is the same. Additionally, $\Sigma^{e}=\mathrm{diag}\left(\sigma^{e}\right)R\mathrm{diag}\left(\sigma^{e}\right)$ and $\Sigma^{p}=\mathrm{diag}\left(\sigma^{p}\right)R\mathrm{diag}\left(\sigma^{p}\right)$, where the correlation matrix R is the same for both the existing and the prospective costs and $\sigma^{e}={\left(\sigma_{i}^{e}\right)}_{N\times 1},\sigma^{p}={\left(\sigma_{i}^{p}\right)}_{N\times 1}$ are standard deviation vectors.

Defining $C={\left(C^{e^{\prime }}\;C^{p^{\prime }}\right)}^{\prime }$, it follows that

(4) $$C|\mu ,\Sigma ∼N\left(\mu ,\Sigma \right),$$

where

(5) $$\mu ={\left(\mu^{e^{\prime }}\;\mu^{p^{\prime }}\right)}^{\prime }\;\mathrm{and}\;\Sigma =\left(\begin{array}{cc} \Sigma^{e} & \mathrm{diag}\left(\sigma^{e}\right)R\mathrm{diag}\left(\sigma^{p}\right)\\ \mathrm{diag}\left(\sigma^{e}\right)R\mathrm{diag}\left(\sigma^{p}\right) & \Sigma^{p} \end{array}\right).$$

The portfolio weights are the proportions of the total budget allocated in each technology. The allocation vectors in the existent and prospective technologies are denoted by $\omega^{e}={\left(\omega_{i}^{e}\right)}_{N\times 1}$ and $\omega^{p}={\left(\omega_{i}^{p}\right)}_{N\times 1}$, respectively. Naturally, $0\leq \omega_{i}^{e}\leq 1$, ∀i=1,2,…,N; $0\leq \omega_{i}^{p}\leq 1$, ∀i=1,2,…,N; and

(6) $$\sum_{i=1}^{N}(\omega_{i}^{e}+\omega_{i}^{p})=1.$$

Defining $\omega ={\left(\omega^{e^{\prime }}\;\omega^{p^{\prime }}\right)}^{\prime }$, we denote by Ω the set of admissible electricity generation mix so that we must have ω∈Ω. The set Ω will represent constraints like Equation (6), $\omega^{\prime }1_{2N}=1$ ($1_{2N}$ is a 2N×1 vector of ones), and minimum and/or maximum values for the allocations ($\omega_{\min}\leq \omega$ and/or $\omega \leq \omega_{\max}$). Using the ω definition, the total cost of the portfolio is given by

(7) $$C=\omega^{\prime }C.$$

Using the previous Equation (7), the expected cost of the portfolio is given by

(8) $$E\left[C\right]=\omega^{\prime }\mu$$

and the variance of the portfolio is given by

(9) $$\mathrm{Var}\left[C\right]=\omega^{\prime }\Sigma \omega .$$

For the case in which the vector of expected costs μ and the covariance matrix Σ are known, three kinds of mean-variance problems are usually considered in the MPT literature (for the details, see [20]). In the following, we translate the three kinds of mean-variance problems to the electricity generation planning context. The first approach minimizes the variance of the costs conditional on a target maximum expected cost *c*. The target maximum expected cost $c\in {ℜ}_{+}$ is provided by the electricity energy policy planner, which represents the maximum allowable expected energy cost. Formally, the problem is written as follows:

(10) $$\min_{\omega }\omega^{\prime }\Sigma ,\omega$$

(11) $$s.t.\;\omega^{\prime }\mu \leq c,\;\omega \in \Omega .$$

The second approach, a dual form of the first approach, minimizes the expected cost conditional on a maximum value $s^{2}$ for the variance of the costs. The value $s^{2}\in {ℜ}_{+}$, provided by the policy planner, represents the maximum value that the variance of the cost could achieve. Formally, the problem is written as follows:

(12) $$\min_{\omega }\omega^{\prime }\mu ,$$

(13) $$s.t.\;\omega^{\prime }\Sigma \omega \leq s^{2},\;\omega \in \Omega .$$

The third approach minimizes a combination of the expectation and variance of the costs, weighted by a risk aversion parameter λ>0. Higher value of λ indicates a greater risk aversion. Formally, the problem is written as follows:

(14) $$\min_{\omega \in \Omega }\omega^{\prime }\mu +\lambda \omega^{\prime }\Sigma \omega .$$

Considering linear constraints and known expected costs μ and covariance matrix Σ, the solution of the previous optimization problem is trivially obtained using any quadratic programming solver. Actually, it is possible to rewrite the previous optimization problem as follows:

(15) $$\min_{\omega \in \Omega }E\left[\varphi \left(C\right)\right]\equiv \min_{\omega \in \Omega }E\left[\varphi \left(\omega^{\prime }C\right)\right]\equiv \min_{\omega \in \Omega }\int \varphi \left(\omega^{\prime }c\right)p\left(c|\mu ,\Sigma \right)dc,$$

where φ is the quadratic cost function such that

(16) $$E\left[\varphi \left(C\right)\right]\equiv \omega^{\prime }\mu +\lambda \omega^{\prime }\Sigma \omega$$

and $p\left(c|\mu ,\Sigma \right)$ is the multivariate Gaussian or normal probability density function with mean μ and covariance matrix Σ. In the MPT context, the approximation of the investor utility function using a quadratic function was shown to be exact when the input data is elliptically distributed [37]. For instance, elliptical distribution includes the normal, Student’s *t* and Levy distributions.

## 3. Classical-Equivalent Bayesian Mean-Variance Approach

In terms of modeling, the Bayesian approaches, compared with the approaches from the last section, address estimation risk from a different angle. In place of treating the unknown parameters as constants, they are considered random. Additionally, the belief or prior knowledge of the specialist about the input parameters is combined with the observed data. The Bayesian models provide an entire distribution of predicted costs that explicitly consider the estimation and predictive uncertainty [21].

The predictive, posterior or updated density of the unknown parameters μ and Σ, according to the Bayes’ theorem, is given by

(17) $$p\left(\mu ,\Sigma |c_{1},\ldots ,c_{T}\right)\propto L\left(\mu ,\Sigma |c_{1},\ldots ,c_{T}\right)\pi \left(\mu ,\Sigma \right),$$

where $c_{1},\ldots ,c_{T}$ are recorded observations; $L\left(\cdot |\cdot \right)$ is the likelihood function; and $\pi \left(\cdot \right)$ is the prior distribution. Particularly, the likelihood function is given by

(18) $$L\left(\mu ,\Sigma |c_{1},\ldots ,c_{T}\right)\propto {\left|\Sigma \right|}^{-\frac{T}{2}}\exp\left[-\frac{1}{2}\sum_{i=1}^{T}{\left(c_{i}-\mu \right)}^{\prime }\Sigma^{-1}\left(c_{i}-\mu \right)\right],$$

where $\left|\Sigma \right|$ is the determinant of the covariance matrix.

Using the predictive density of the unknown parameters μ and Σ from Equation (17), it is possible to obtain the predictive density of the costs as

(19) $$p\left(c|c_{1},\ldots ,c_{T}\right)\propto \int p\left(c|\mu ,\Sigma \right)p\left(\mu ,\Sigma |c_{1},\ldots ,c_{T}\right)d\mu d\Sigma .$$

Then, using the predictive density of the costs in the optimization problem from Equation (15), the Bayesian optimization problem is defined by

(20) $$\arg\min_{\omega \in \Omega }\int \varphi \left(\omega^{\prime }c\right)p\left(c|c_{1},\ldots ,c_{T}\right)dc.$$

In the following subsections, we present the predictive distributions using improper and proper priors for the unknown parameters μ and Σ.

### 3.1. Improper Prior Case

In some cases, our prior beliefs are vague and thus difficult to express into an informative prior. Consequently, we would like to still consider the uncertainty of the model parameters without impacting them with any prior belief. The improper priors, also called non-informative, diffuse or vague priors, are employed to that end. We consider the case when the investor is uncertain about the distribution of both parameters, μ and Σ, and has no particular prior knowledge of them. This case is modeled using an improper prior, which is typically chosen to be the Jeffreys’ prior [38]

(21) $$\pi \left(\mu ,\Sigma \right)\propto {\left|\Sigma \right|}^{-\frac{\left(2N+1\right)}{2}},$$

where μ and Σ are considered independent in the prior, and μ is not restricted as to the values it can take. The prior is non-informative in the sense that only changes in the data exert an influence on the predictive distribution of the parameters. When the sample mean, $\hat{\mu }$, and sample covariance matrix, $\hat{\Sigma }$, are given, it is straightforward to verify that the predictive distribution of the costs is a multivariate Student’s *t*-distribution (for the complete derivation of the following result, see [20] or [21])

(22) $$C|\tilde{\mu },\tilde{\Sigma }∼t_{T-2N}(\tilde{\mu },\tilde{\Sigma }),T-2N\geq 2,$$

where the predictive mean and covariance matrix are, respectively,

(23) $$\tilde{\mu }=\hat{\mu }\mathrm{and}\tilde{\Sigma }=\frac{\left(1+T^{-1}\right)\left(T-1\right)}{T-2N-2}\hat{\Sigma }.$$

Here, the predictive covariance matrix represents the sample covariance scaled up by a factor, reflecting the estimation risk. For a given number of technologies *N*, $\tilde{\Sigma }$ becomes closer to $\hat{\Sigma }$ as more historical data are available. Actually, when *N* is fixed and T→∞, we have $\tilde{\Sigma }\to \hat{\Sigma }$. On the other hand, with a fixed number of historical observations *T*, increasing the number of technologies *N* respecting the constraint T−2N−2>0, leads to higher absolute numerical values for the covariance matrix and estimation risk, since the relative amount of available data decreases. In practice, there are relevant information coming from specialists on energy costs. Consequently, in the next subsection, we present a study with proper priors.

To conclude, the classical-equivalent Bayesian optimization problem for electricity generation planning for the improper prior case is given by

(24) $$\min_{\omega \in \Omega }\omega^{\prime }\tilde{\mu }+\lambda \omega^{\prime }\tilde{\Sigma }\omega .$$

### 3.2. Proper Prior Case

In the proper prior case, the specialists have informative beliefs about the mean and covariance of technology costs. Particularly, in this subsection, we adopt conjugate priors because it is an algebraic convenience producing a closed expression for the posterior. Using a similar approach common in the investment portfolio allocation context [20,21], the conjugate prior for the mean vector of the normal distribution (conditional on Σ) is taken to be the multivariate normal while the conjugate prior for the unknown covariance matrix of the normal distribution is taken to be the inverse Wishart distribution:

(25) $$\mu |\Sigma ∼N\left(\eta ,\frac{1}{\tau }\Sigma \right),\Sigma ∼W^{-1}\left(\Psi ,\nu \right),$$

where η is the vector of expected costs based on the specialist experience, $\tau \in {ℜ}_{+}$ represents the confidence strength the specialist places on the value of η, Ψ is the covariance matrix based on the specialist experience, and ν∈ℜ represents the degrees of freedom of the inverse Wishart distribution reflecting the confidence about Ψ. Lower values of τ and ν indicates higher uncertainty about η and Ψ, respectively.

As in the improper prior case, the predictive distribution of the costs is a multivariate Student’s *t*-distribution (for the complete derivation of the following result, see [20] or [21])

(26) $$C|\overset{˘}{\mu },\overset{˘}{\Sigma }∼t_{T-2N}(\overset{˘}{\mu },\overset{˘}{\Sigma }),T-2N\geq 2,$$

where the predictive mean and covariance matrix are, respectively,

(27) $$\overset{˘}{\mu }=\frac{\tau }{T+\tau }\eta +\frac{T}{T+\tau }\hat{\mu }$$

and

(28) $$\overset{˘}{\Sigma }=\frac{T+1}{T\left(\nu +2N-1\right)}\left[\Psi +\left(T-1\right)\hat{\Sigma }+\frac{T\tau }{T+\tau }\left(\eta -\hat{\mu }\right){\left(\eta -\hat{\mu }\right)}^{\prime }\right].$$

We notice that the predictive mean $\overset{˘}{\mu }$ is a weighted average of the prior mean, η, and the sample mean, $\hat{\mu }$. In other words, the sample mean is shrunk toward the prior mean. Actually, the predictive mean and predictive covariance matrix are not proportional to the sample estimates. The improper prior case is suitable to use when we do not suspect that the sample mean or sample covariance matrix contain considerable estimation errors. Alternatively, the proper prior case is better when the planner believes that, in the future, the expectation and covariance matrix of the costs will differ substantially from the historical ones.

To conclude, the classical-equivalent Bayesian optimization problem for electricity generation planning for the proper prior case is given by

(29) $$\min_{\omega \in \Omega }\omega^{\prime }\overset{˘}{\mu }+\lambda \omega^{\prime }\overset{˘}{\Sigma }\omega .$$

## 4. Results

In this section, we present a application to illustrate the classical-equivalent Bayesian approaches. In Table 1, we reproduce from [35] the means and standard deviations of costs for maintenance of existing plants and building of new plants for different energy generation technologies: (1) gas; (2) coal; (3) nuclear; (4) fuel oil; (5) biomass; (6) large hydropower; (7) wind; and (8) low-impact or small hydropower. Additionally, in Table 2, we also reproduce from [35] the correlation matrix of the technologies considering the fuel costs. Since the correlation matrix is symmetric, we do not repeat the elements. In [35], the data was obtained using the Levelized Busbar Cost (LBC) methodology (for instance, see [4]). LBC is a valuation technique that calculates the costs over the electric plants’ useful lifetimes and averages them to yield a total production cost. For the purpose of our application, we consider the data from Table 1 and Table 2 as the sample estimates of ${\hat{\mu }}^{e}$, ${\hat{\mu }}^{p}$, ${\hat{\sigma }}^{e}$, ${\hat{\sigma }}^{p}$ and $\hat{R}$.

**Figure 1:**
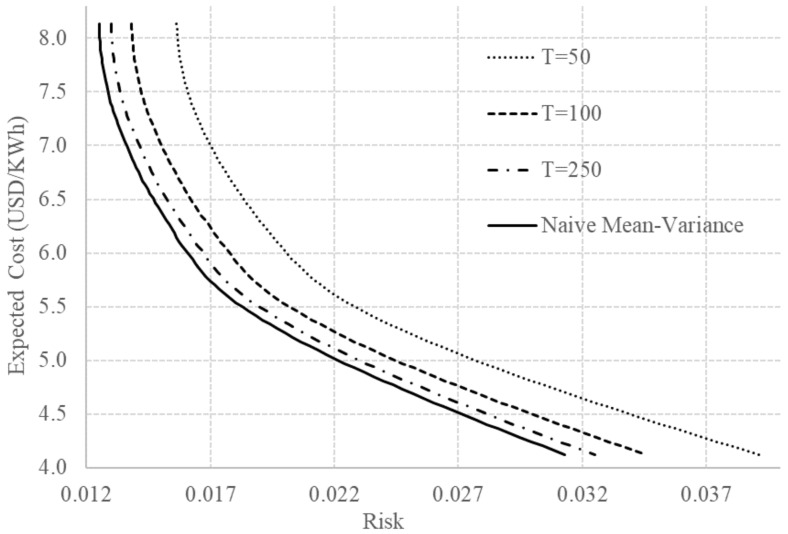
Efficient frontiers using naive and classical-equivalent Bayesian approaches for the improper prior case for some values of *T*.

**Figure 2:**
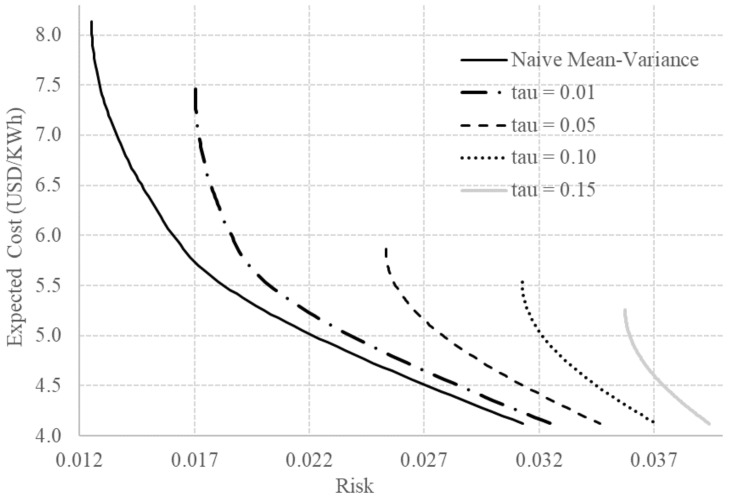
Efficient frontiers using naive and classical-equivalent Bayesian approaches for the proper prior case for some values of τ.

The naive mean-variance efficient frontier obtained using $\hat{\mu }$ and $\hat{\Sigma }$ is presented in Figure 1 and Figure 2 (repeated in the two graphics). The efficient frontier represent the set of all optimal choices. It is important to notice that the portfolios above the efficient frontier are realizable but inefficient and the portfolios below the efficient frontier are unrealizable. On the other hand, in the MPT context, since the random variables are the returns instead of the costs, the portfolios below the efficient frontier are realizable but inefficient, and the portfolios above the efficient frontier are unrealizable. It is fundamental to highlight the differences between the set of realizable portfolios in the two contexts to avoid misinterpretations. In addition, it is also important to notice that the efficient frontier for the costs is always convex while the efficient frontier for the returns is always concave.

In the MPT context, the efficient frontier is calculated without considering the risk-free asset [20]. The risk-free asset is the one with a certain future return. The identification of the risk-free asset depends on the context of interest. For example, in the United States, the treasury bills (T-bills) are considered the risk-free asset because they are backed by the government. Analogously, in the energy planning context, the efficient frontier must be calculated without considering the risk-free energy generation technologies. The existence of risk-free technologies also depends on the context of interest. For example, government backed subsidized energy generation technologies could be considered risk-free technologies. After obtaining the efficient frontier, following the MPT procedure, the risk-free technologies must be linearly combined with the efficient portfolios to obtain new optimal portfolios [39].

In the improper prior case, illustrated in Figure 1, the efficient frontier changes depending on the value of *T*. As mentioned, the predictive covariance of the improper case is the sample covariance scaled up by a factor that approaches to one when *T* increases. Obviously, we do not have *T* here representing the actual size of the sample used in the estimation. Actually, for us, *T* is not only a proxy to the size of the sample used in the estimation but also the degree of confidence the planner has on the estimations based only on historical data. Consequently, decreasing the value of *T* shifts the efficient frontier to the right. The same shift to the right was observed in [19] using the robust mean-variance optimization with uncertainty sets when decreasing the degree of confidence the planner has on the estimations. In other words, the robust mean-variance optimization and our improper prior case include the uncertainty of the estimations in the electricity planning process. However, we highlight the fact that the robust mean-variance optimization is computationally more expensive than our approach because it requires several optimizations to cover all the uncertainty set. Our improper prior case only requires a single optimization.

In the proper prior case, the hyperparameters η and Ψ represent the prior information of the specialist about the expected value and covariance matrix of the technology costs, respectively. Since we do not have such parameters for the situation described in [35], we assume, for illustration purposes, that η and Ψ are obtained increasing by 10% the vectors $\hat{\mu }$, $\hat{\sigma^{e}}$ and $\hat{\sigma^{p}}$. Unfortunately, specialist priors are not publicly available. Consequently, in the proper prior case, the application is just a toy problem for illustration purposes. In Figure 2, we present the obtained efficient frontiers for different values of τ with T=50 and ν=34. Noticeably, the resulting efficient frontiers are not simple shifts of the naive mean-variance frontier. Consequently, as mentioned, the informative proper prior case is most suitable to use than the improper prior case when the planner believes that in the future the costs will differ substantially from the historically estimated ones. In this section, the objective was to show the flexibility and the potential of applicability of the Bayesian approach to include not only the estimation uncertainty, but also the specialist information in the energy planning process.

## 5. Conclusions

In this paper, we introduce the use of the classical-equivalent Bayesian mean-variance optimization in the electricity generation planning. We illustrate the application of the approach using improper and proper priors. Comparing with the existent robust approach to electricity portfolio selection, the classical-equivalent Bayesian approach has the advantage of not only dealing with the estimation uncertainty, but also considering the prior information of the specialists in the planning process. Particularly, in the proper prior case, we have assumed that the covariance matrix of the expected value of the costs are proportional to the covariance matrix of the costs. In practice, the assumption is not necessarily valid. For future research, we suggest the investigation of changing the proper priors to give more flexibility to the electricity generation planner and the use of real priors from the specialists. The real prior distributions are not necessarily conjugate for the likelihood function. Consequently, a closed form of posterior distribution may not exist. In this case, it is necessary to approximate the posterior distribution using, for instance, Markov chain Monte Carlo method via the Metropolis–Hastings algorithm [40].

## Figures and Tables

**Table 1:** The means and standard deviations of costs for existing plants and prospective ideas of building new plants for different energy generation technologies from [35] (values are in cents of USD/kWh).

Energy Generation Technology	${\hat{\mu }}_{i}^{e}$	${\hat{\mu }}_{i}^{p}$	${\hat{\sigma }}_{i}^{e}$	${\hat{\sigma }}_{i}^{p}$
gas	09.9010	09.2770	0.1500	0.1500
coal	11.5560	11.1180	0.1125	0.1187
nuclear	10.1260	10.0110	0.0625	0.1500
fuel oil	19.0980	16.4680	0.2250	0.2188
biomass	14.0390	13.4560	0.0813	0.0875
large hydropower	04.1200	05.0240	0.0313	0.2062
wind	10.9860	10.4440	0.0250	0.1187
small hydropower	06.8850	06.9090	0.0187	0.1187

**Table 2:** The correlations of the fuel costs between different energy generation technologies from [35].

Energy Generation Technology	1.	2.	3.	4.	5.	6.	7.	8.
1. gas	01.00							
2. coal	00.47	01.00						
3. nuclear	00.06	00.12	01.00					
4. fuel oil	00.49	00.27	00.08	01.00				
5. biomass	−0.44	−0.38	−0.22	−0.17	01.00			
6. large hydropower	00.00	00.00	00.00	00.00	00.00	01.00		
7. wind	00.00	00.00	00.00	00.00	00.00	00.00	01.00	
8. small hydropower	00.00	00.00	00.00	00.00	00.00	00.00	00.00	1.00

## Abbreviations

The following abbreviations are used in this manuscript:

MPT	Modern Portfolio Theory
USD	United States Dollar
